# When 2D is not enough: 3D transesophageal echocardiography revealing a multifenestrated atrial septal defect

**DOI:** 10.1186/s44348-026-00103-7

**Published:** 2026-10-22

**Authors:** Jaime Andrés Nieto, David Aparicio, Susan Julieth Flórez Rubio

**Affiliations:** 1 Department of Cardiology, Clínica Universitaria Colombia, Bogotá, Colombia; 2 Department of Internal Medicine, Clínica Universitaria Colombia, Bogotá, Colombia; 3 Fundación Universitaria Sanitas, Clínica Universitaria Colombia, Bogotá, Colombia

**Keywords:** Heart Septal Defects, Atrial Echocardiography, Transesophageal Imaging, Three-Dimensional Heart Defects, Congenital Cardiac Catheterization Hemodynamics

## Background

Fenestrated atrial septal defects represent a complex anatomical variant characterized by multiple perforations within a thinned interatrial septum. Although frequently asymptomatic, these defects may remain undetected or be underestimated by conventional 2D echocardiography [1]. A 3D transesophageal echocardiography (TEE) provides enhanced spatial visualization, allowing accurate characterization of the number, distribution, and spatial relationship of multiple fenestrations [2]. In multifenestrated ostium secundum atrial septal defects (ASDs), this information extends beyond diagnostic confirmation and represents a key element in assessing closure feasibility and determining the most appropriate therapeutic approach, including transcatheter versus surgical closure [3]. Accurate anatomical characterization may therefore contribute to timely management aimed at preventing the progression of chronic right-sided volume overload and its long-term hemodynamic consequences.

## Case presentation

A 40-year-old woman with a history of obesity and dyslipidemia was referred for further evaluation after a screening transthoracic echocardiogram performed at an outside institution raised suspicion of an interatrial shunt. The patient was clinically asymptomatic. TEE was subsequently performed to achieve detailed anatomical characterization of the defect.

**Fig. 1:**
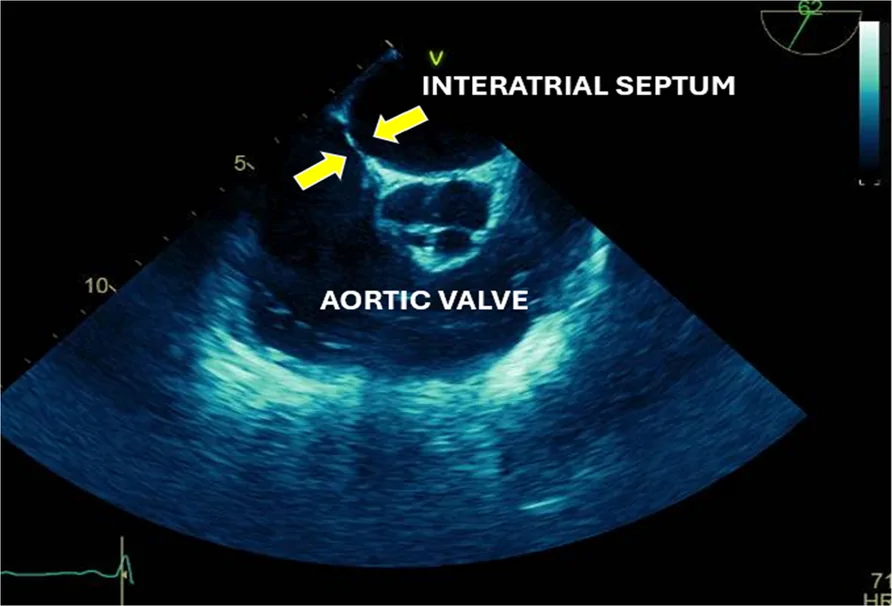
Two-dimensional transesophageal echocardiography performed following sedation and local anesthesia using a matrix-array 3D transesophageal probe (Philips). In this initial mid-esophageal view at 60°, a thinned interatrial septum with an apparent focal discontinuity (arrows) is observed, suggestive of an interatrial communication. The aortic valve is visualized inferiorly as an anatomical reference

Following sedation and local anesthesia, TEE was performed using a matrix-array 3D transesophageal probe (Philips). Initial 2D assessment from the mid-esophageal view at 60° demonstrated a thinned interatrial septum with an apparent discontinuity suggestive of an interatrial communication (Fig. 1).

**Fig. 2:**
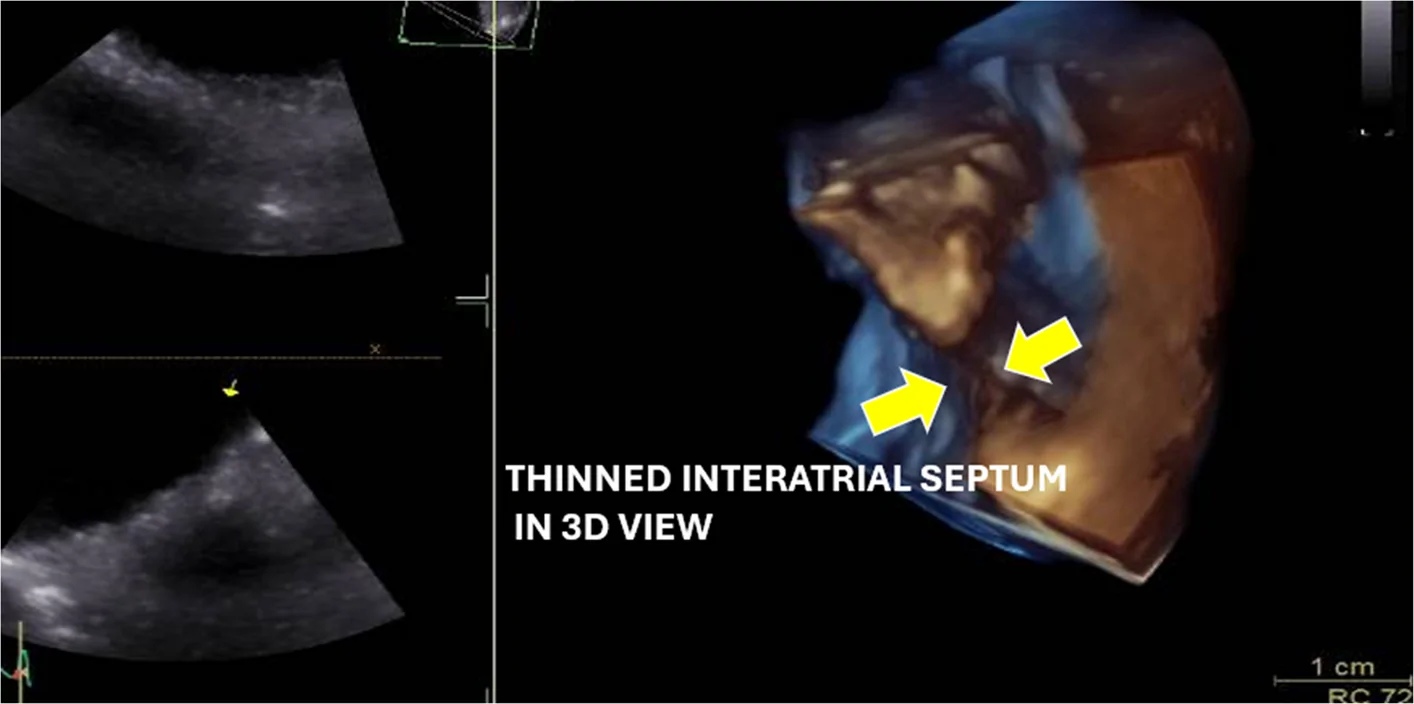
Three-dimensional reconstruction of the interatrial defect (84° view) obtained using X-plane imaging and 3D zoom. Discontinuity of the interatrial septum (arrows) is demonstrated, confirming the focal thinning and structural defects suggested by the initial 2D assessment

**Fig. 3:**
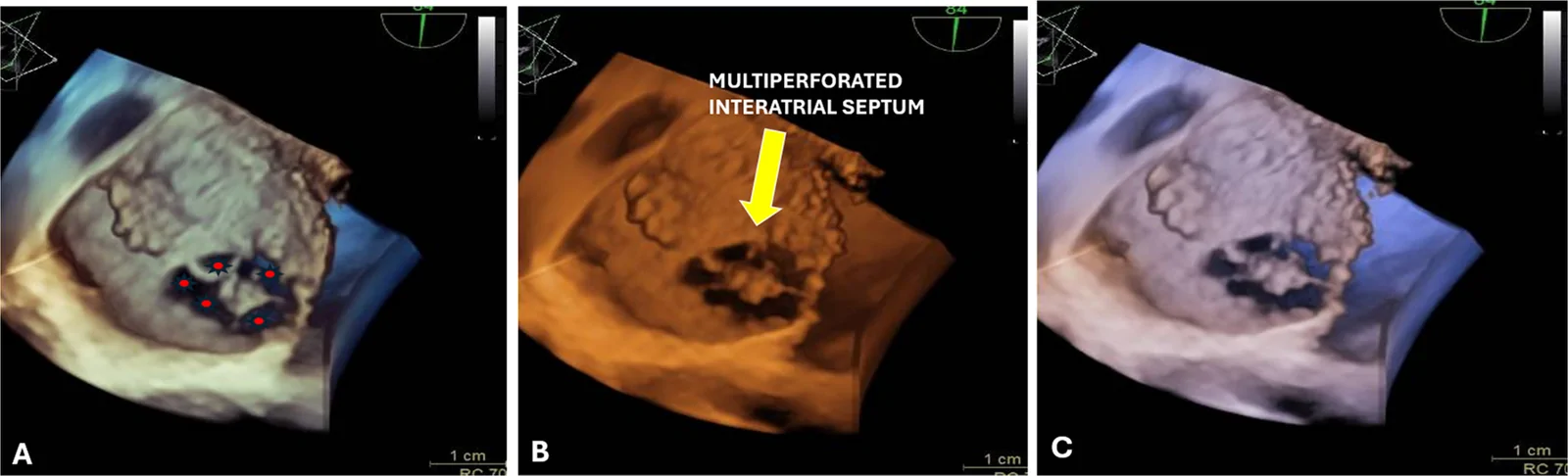
Three-dimensional multiview reconstruction using colorization and parametric adjustment, providing high-definition visualization of the interatrial septum. **A** The *en face* view using standard 3D rendering to visualize the overall anatomy. **B** The fenestrated region (arrow) using a depth-based color map is highlighted, clearly outlining the five distinct defects in a cogwheel-like configuration. **C** An alternative illumination model that enhances tissue depth and the spatial relationship between the perforations is provided. Together, these views confirm an overall fenestrated region spanning 1.3 cm, corresponding to approximately one-third of the total diameter of the interatrial septum

To further define the anatomy, X-plane imaging and 3D zoom were used (Fig. 2). Multiview parametric adjustment, colorization, and modified orthogonal views provided high-definition *en face* visualization of the interatrial septum, confirming a multifenestrated ostium secundum ASD. Five distinct defects were identified in a characteristic cogwheel-like configuration, with an overall transverse diameter of 1.3 cm, corresponding to approximately one-third of the total diameter of the interatrial septum (Fig. 3).

Hemodynamic assessment with color Doppler confirmed a left-to-right interatrial shunt. Despite the absence of clinical symptoms, the study demonstrated clear structural repercussions on the right-sided cardiac chambers. The right atrial area was 26 cm^2^, and the right ventricle showed mild basal dilatation, with a basal diameter of 4.2 cm, a mid-cavity diameter of 3.4 cm, and a longitudinal diameter of 6.3 cm. Right ventricular systolic function was qualitatively preserved. The calculated pulmonary-to-systemic flow ratio (Qp/Qs) was 1.5:1, indicating increased pulmonary relative to systemic blood flow. Pulmonary artery systolic pressure was estimated at 25 mmHg based on a peak tricuspid regurgitation velocity of 2.4 m/sec.

Clinical management was based on the correlation between the anatomical findings and their hemodynamic impact. Although the patient remained completely asymptomatic, the presence of right ventricular volume overload, evidenced by a Qp/Qs of 1.5:1 and mild basal right ventricular dilatation, supported consideration of ASD closure rather than clinical surveillance alone, with the aim of preventing progressive adverse right-sided cardiac remodeling and its long-term hemodynamic consequences.

Accordingly, the patient was referred for multidisciplinary heart team evaluation to assess and plan ostium secundum ASD closure. However, following the initial diagnosis, the patient elected to continue her clinical care and procedural follow-up at an outside institution; therefore, information regarding the definitive closure strategy and subsequent clinical outcomes was not available. In this setting, 3D TEE provided clinically relevant anatomical information by demonstrating that the five fenestrations were distributed within an overall region measuring 1.3 cm and by defining their spatial relationship within the interatrial septum. This detailed anatomical characterization is particularly relevant when assessing the feasibility of transcatheter closure, including whether complete coverage could potentially be achieved with a single occluder device without significant residual shunting or whether the complexity of the septal anatomy would favor an alternative closure strategy.

## Discussion

The evaluation and transcatheter closure of a multifenestrated ASD require meticulous planning and pose significant technical challenges that are not typically encountered with isolated defects [1]. Major considerations when assessing these anatomical variants for closure include the risk of incomplete coverage of all fenestrations, the possibility of significant residual shunting after the procedure, and the potential need for implantation of either a single or multiple occluder devices [2].

In our patient, 3D TEE overcame the limitations of 2D assessment by accurately demonstrating that the five defects, arranged in a cogwheel-like configuration, were clustered within an overall diameter of 1.3 cm [3]. Precise assessment of the number, spatial distribution, and relationship of the fenestrations to the surrounding interatrial septal tissue provided clinically relevant anatomical information that may be underestimated or incompletely defined by 2D TEE alone [1, 2]. These findings provided relevant anatomical information for the heart team to assess the feasibility of transcatheter closure, including the potential for complete coverage of the fenestrated region with a single occluder device versus the need for an alternative closure strategy [2].

**Fig. 4:**
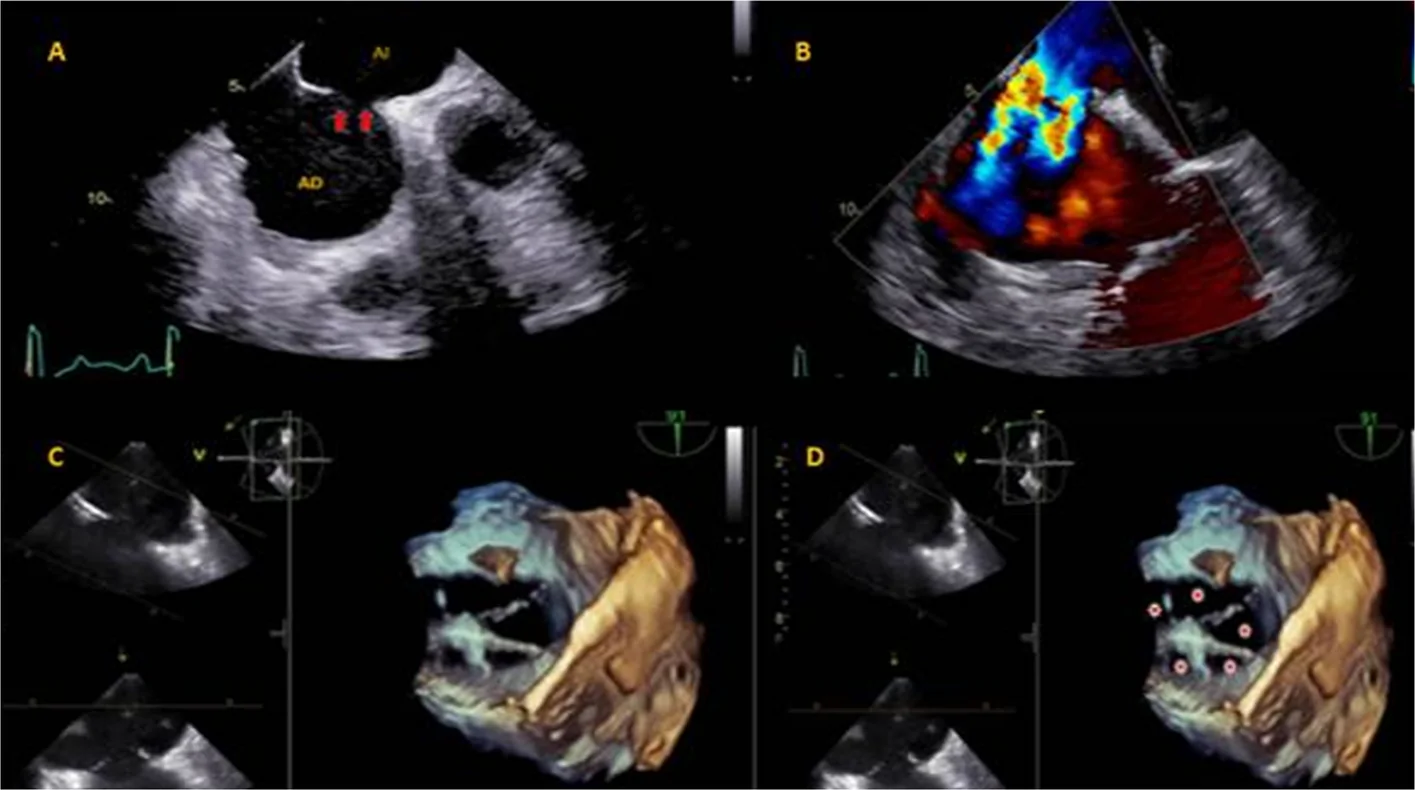
Two-dimensional transesophageal echocardiography with color Doppler (**A**, **B**) and 3D reconstruction (**C**, **D**) from a case of multifenestrated atrial septal defect previously published by the authors. The panels illustrate multiple flow jets across the interatrial septum (color mosaic in panel B), spatially correlating the sites of interatrial shunting with the fenestrated anatomy (asterisks in panel D). Reproduced from Nieto-Zárate et al. [3], available under the Creative Commons license (CC BY 4.0)

As previously illustrated in a case published by Nieto-Zárate et al. [3] (Fig. 4), 3D TEE can correlate complex interatrial septal anatomy with the corresponding multiple flow jets visualized on color Doppler, further supporting its role in clinical decision-making. The incorporation of 3D imaging into the evaluation of multifenestrated ASDs may help avoid underestimation of the anatomical extent of the defect, reduce diagnostic uncertainty, and optimize procedural planning for complex structural heart interventions [1–3].

## Conclusions

In this asymptomatic patient, 3D TEE enabled precise spatial characterization of five fenestrations arranged in a cogwheel-like configuration within a region spanning 1.3 cm, a complex anatomy that was not fully characterized by conventional 2D imaging. This detailed anatomical delineation, together with evidence of hemodynamic repercussion, including a Qp/Qs of 1.5:1 and mild right ventricular dilatation, provided clinically relevant information for assessing the feasibility of defect closure and guiding referral for definitive therapeutic evaluation. This case highlights the incremental value of 3D TEE in translating complex septal anatomy into information directly relevant to therapeutic planning.

## Data Availability

The data supporting the findings of this case report are not publicly available due to patient privacy and confidentiality. All relevant data necessary to interpret the case are included in the article.

## Abbreviations

ASD: Atrial septal defects
Qp/Qs: Pulmonary-to-systemic flow ratio
TEE: Transesophageal echocardiography
